# Years of life lost by COVID-19 in Portugal and comparison with other European countries in 2020

**DOI:** 10.1186/s12889-021-11128-6

**Published:** 2021-06-02

**Authors:** André Vieira, Vasco Peixoto Ricoca, Pedro Aguiar, Paulo Sousa, Carla Nunes, Alexandre Abrantes

**Affiliations:** 1 NOVA National School of Public Health, Public Health Research Centre, Universidade NOVA de Lisboa, Lisbon, Portugal; 2 Comprehensive Health Research Centre, Universidade NOVA de Lisboa, Avenida Padre Cruz, 1600-560 Lisbon, Portugal

**Keywords:** Coronavirus, Years of life lost, Mortality, Pandemics, Disease outbreaks

## Abstract

**Background:**

The impact of the COVID-19 pandemic has been measured in different metrics, mostly by counting deaths and its impact on health services. Few studies have attempted to calculate years of life lost (YLL) to COVID-19 and compare it with YLL due to other causes in different countries.

**Methods:**

We calculated YLL to COVID-19 from week10 to week52 in 2020 for eight European countries by methods defined by the WHO. We calculated excess YLL by subtracting the average YLL from 2017 to 2019 to the YLL in 2020. Our analysis compared YLL to COVID-19 and the excess YLL of non-COVID-19 causes across countries in Europe.

**Results:**

Portugal registered 394,573 cases and 6619 deaths due to COVID-19, accounting for 25,395 YLL in just 10 months. COVID-19 was responsible for 6.7% of all deaths but accounted for only 4.2% of all YLL. We estimate that Portugal experienced an excess of 35,510 YLL (+ 6.2%), of which 72% would have been due to COVID-19 and 28% due to non-COVID-19 causes. Spain, Portugal, and the Netherlands experienced excess YLL to non-COVID-19 causes. We also estimated that Portugal experienced an excess of 10,115 YLL due to cancer (3805), cardiovascular diseases (786) and diseases of the respiratory system (525).

**Conclusion:**

COVID-19 has had a major impact on mortality rates in Portugal, as well as in other European countries. The relative impact of COVID-19 on the number of deaths has been greater than on the number of YLL, because COVID-19 deaths occur mostly in advanced ages.

**Supplementary Information:**

The online version contains supplementary material available at 10.1186/s12889-021-11128-6.

## Background

In 2020, the COVID-19 pandemic spread around the world, causing serious effects on populations [1]. It is a serious threat due to its high transmissibility and overall lethality (1.7% in Portugal, 2.1% in Europe and 2.2% worldwide as of 27 December 2020, not considering under-ascertainment) [1, 2]. This is only comparable to the Spanish flu in the year 1918, for which worldwide lethality is estimated to have been above 2.5% [3].

COVID-19 affects elderly people more severely [4]. In Portugal, there have been relatively few deaths in patients under the age of 50, accounting for only 1.2% of the total number of recorded deaths. On 27 December 2020, around 67.7% of COVID-19 deaths were recorded among people aged 80+ years and 31.2% among those aged 50 to 79 years of age [2].

The Centers for Disease Control and Prevention refers to excess deaths (or excess mortality) as “*the difference between the observed numbers of deaths in specific time periods and expected numbers of deaths in the same time periods*” [5]. EUROMO counted excess mortality across 26 European countries in 2020, including that directly attributed to COVID-19 [6]. However, not all excess deaths were directly caused by COVID-19. Because healthcare systems had to adapt to the overwhelming demands of the pandemic, patients with other common and serious diseases may have not received the level and quality of care that they would have received under normal circumstances [7]. By late May 2020, England and Wales, Italy, and Spain had already reported that 28.8, 32.5 and 61.8% of excess mortality, respectively, was not due to COVID-19, often referring to it as non-COVID-19 excess mortality or collateral mortality [8]. In Portugal, the percentage of non-COVID-19 excess mortality has fluctuated over time, from 51% between March and mid-April, to 92% in July and finally to no excess registered in December 2020 [8–10].

The number of deaths is an imperfect measure of mortality, as it does not provide insight into the age distribution of deaths or how risk levels vary by age. It is equally important to measure premature deaths, in terms of the years expected to live in relation to the person’s life expectancy (LE), and to account for the number of years living with a disability. These two dimensions of the burden of disease can be expressed in terms of potential years of life lost (YLL) due to premature death and to years lived with disability (YLD) [11]. The disability-adjusted life year (DALY) is a known measure of the population’s health that results from their sum [12]. This calculation takes into consideration the age at which a certain death or health condition occurred, which can be estimated for a certain disease or group of diseases, using LE as the reference for the years the person is expected to live. A higher YLL can be due to larger numbers of death, deaths in younger ages, or some combination of the two [13].

Cancer (CA), cardiovascular diseases (CVDs) and diseases of the respiratory system (DRSs) are the most common causes of death in Portugal and tend to kill those affected at younger ages [14]. In 2017, CVDs and CA were the major causes of natural deaths worldwide for those aged 15 to 49 years, accounting for 1.26 million deaths and 1.06 million deaths, respectively [15]. The DRSs were the third cause of death for people aged 50 to 69 years and people aged 70+ years, accounting for 1 million deaths and 3.96 million deaths registered, respectively [15, 16].

The estimation of the number of YLL due to COVID-19 and due to other causes adds to the calculation of excess mortality. Several authors [17, 18] have developed preliminary models for estimating YLL to COVID-19, namely in Italy, where they estimated that the number of YLL per patient due to COVID-19 was about 13 years per male patient and 11 years per female patient.

This study aims to:

- Calculate the burden of COVID-19, in terms of YLL, due to premature deaths for Portugal and for other selected European countries (France, Germany, Italy, the Netherlands, Spain, Sweden, and the United Kingdom);
- Estimate the excess YLL to non-COVID-19 diseases (exc-YLL-non-Cvd) for all countries in the year 2020, after week 10; and
- Estimate the excess YLL in 2020 due to CA, CVDs, and DRSs in the selected countries for the same period.

## Methods

This is an observational, cross-sectional study. Our analysis rests on deaths registered by the Eurostat database for all-cause mortality, from the *Institut National d’Études Démographiques* [19] (INED) and from the Northern Ireland Statistics and Research Agency [20] (NISRA). The calculation method for each objective is explained below:

### YLL

To calculate YLL, we adapted the traditional method, taking into consideration the scarcity of information on causes of mortality by age [12]. The calculations were made as follows:

$$ \mathrm{YLL}={\sum}_{\mathrm{i}}\;{\mathrm{M}}_{\mathrm{i}}\ast \left(\mathrm{LE}\hbox{-} {\mathrm{IRP}}_{\mathrm{i}}\right) $$

Where:

**YLL** = years of life lost;

**M** = number of deaths registered in each age class;

**LE** = life expectancy at birth;

**IRP** = intermediate-range point of the age class; and.

_**i**_ = Age classes with deaths.

Accessible data of the age at the time of deaths by country is grouped in 5- or 10-year ranges, including those due to COVID-19. All calculations for YLL were performed for the counting deaths below the age of 80 years once the values for LE were narrowed to this age, and this was an upper limit recommended by other studies for these calculations [21]. Only data for COVID-19 deaths in Scotland and Northern Ireland were not performed in this way, due to the upper age class being 74 to 85 years, making the inclusion of deaths recorded in this range with values ​​above the LE contributing to some reduction in the final estimate of the YLL for that range.

Descriptive data of deaths by all causes and by COVID-19 for each country can be accessed in Additional file 1.

### Total YLL by COVID-19

To calculate YLL by COVID-19, M_**i**_ was replaced by the number of deaths due to COVID-19 by age. Information was retrieved from the INED [19] for all countries and from the INED and NISRA [20] for the United Kingdom (the INED for Scotland and England and Wales and the NISRA for Northern Ireland). For these calculations, the formula with the latest known LE value for each country was used, without considering gender differentiation (Additional file 1) [22].

To estimate the percentage of YLL by COVID-19 by gender for each country, data about the LEs for males and females were also retrieved for the latest value known (Additional file 1) [22]. Due to a lack of data, this calculation was not possible for Sweden.

### Exc-YLL-non-CVD in 2020 by country

The exc-YLL-non-Cvd for each country for the period covered by the study was calculated using the following formula:

$$ \left(\mathrm{Exc}\right)\ \mathrm{YLL}\ \mathrm{non}\ {\mathrm{Cvd}}_{YLL}= Tota{l}_{YLL2020}- Avera\mathrm{g}{e}_{YLL2017.2019}- COVI{D}_{YLL2020} $$

For comparative purposes, we calculated the rates of YLL by country population in order to achieve YLL per 10,000 habitants for each country [23].

We also calculated the ratio for each country between the Exc-YLL-non-CVD/YLL COVID-19 for each country.

To estimate the exc-YLL-non-Cvd by country, we estimated the average number of YLL expected in 2020, based on the mortality registered in 2017–2019, and compared it with that registered in 2020. We retrieved the mortality data by all-causes and by age between 2 March (week 10) and 27 December (week 52) for the years 2017, 2018, and 2019 from the Eurostat database [24]. March was chosen because it was when Europe surpassed 500 deaths registered by COVID-19 [1]. When the data for 27 December were not available, data were retrieved from the closest day available. An exception was made for Italy, where analyses were only performed until week 49, as the data was available on the day of this study. The same procedure was performed for the year 2020, to achieve the total YLL in 2020, for the same weeks in consideration. All estimations were performed with the latest LE values known for both genders as mentioned above.

Then, we analysed the correlation between the YLL by COVID-19 and the exc-YLL-non-Cvd found in the selected countries through Pearson’s correlation, with a statistical significance of 95%.

### Exc-YLL-non-Cvd for disease groups in 2020 by country

Because mortality statistics by age and by specific causes (i.e., the International Classification of Diseases [25]) for some countries were only available until 2016 [26], we estimated the YLL for each disease in 2020 based on the percentage data from previous years (2012–2016). We also included data from 28 European Union countries for comparative purposes.

First, we calculated the percentage of YLL attributed to each disease in each year using the data between 2012 and 2016 through the following formula:

$$ Average\kern0.5em \%\kern0.5em of\kern0.5em YLL\kern0.5em for\kern0.5em a\kern0.5em disease=\frac{\frac{\mathrm{YLL}\kern0.5em \mathrm{for}\kern0.5em \mathrm{a}\kern0.5em \mathrm{disease}\kern0.5em 2012}{\mathrm{All}\kern0.5em \mathrm{YLL}\kern0.5em 2012}+\left(\dots \right)+\frac{\mathrm{YLL}\kern0.5em \mathrm{for}\kern0.5em \mathrm{a}\kern0.5em \mathrm{disease}\kern0.5em 2016}{\mathrm{All}\kern0.5em \mathrm{YLL}\kern0.5em 2016}}{5} $$

The LE used for the YLL calculation was the value of each country in each year (Additional file 1) [22]. Then, we calculated the percentage of YLL from premature deaths for the selected group of diseases per year in relation to all YLL in that year. Thereby, we estimated the average burden of each group of selected diseases (CA, CVDs, and DRSs) over those 5 years.

Finally, we estimated the excess YLL for each group of diseases in 2020 through the equation:

$$ Excess\  YLL\  for\ a\  diseas{e}_{2020}= Exc\  YLL\  non\  Cvd\ast Average\% of\  YLL\  for\ a\  diseas e $$

We only presented the results when exc-YLL-non-Cvd had a positive value and assumed that the proportion of YLL by disease remained the same across the years.

## Results

### Burden of COVID-19 in terms of YLL

Between 2 March and 27 December 272,020, Portugal registered 394,573 cases and 6619 deaths due to COVID-19 [2]. Given the age at the time of death, these deaths accounted for 25,395 YLL to COVID-19 in just 10 months (Table 1). During that period, the total YLL due to premature deaths was 611,104 YLL. While COVID-19 was responsible for 6.7% of all deaths, it accounted for only 4.2% of YLL during this period.

**Table 1:** Number of Deaths and Years of Life Lost in Portugal during the COVID-19 Pandemic (2 March-27 December 2020)

	COVID-19	Non-COVID-19	Total
N° of deaths	6619	92,749	99,368
%	6.7	93.3	100.0
N° of YLL	25,395	585,710	611,104
%	4.2	95.8	100

*YLL* years of life lost

**Fig. 1:**
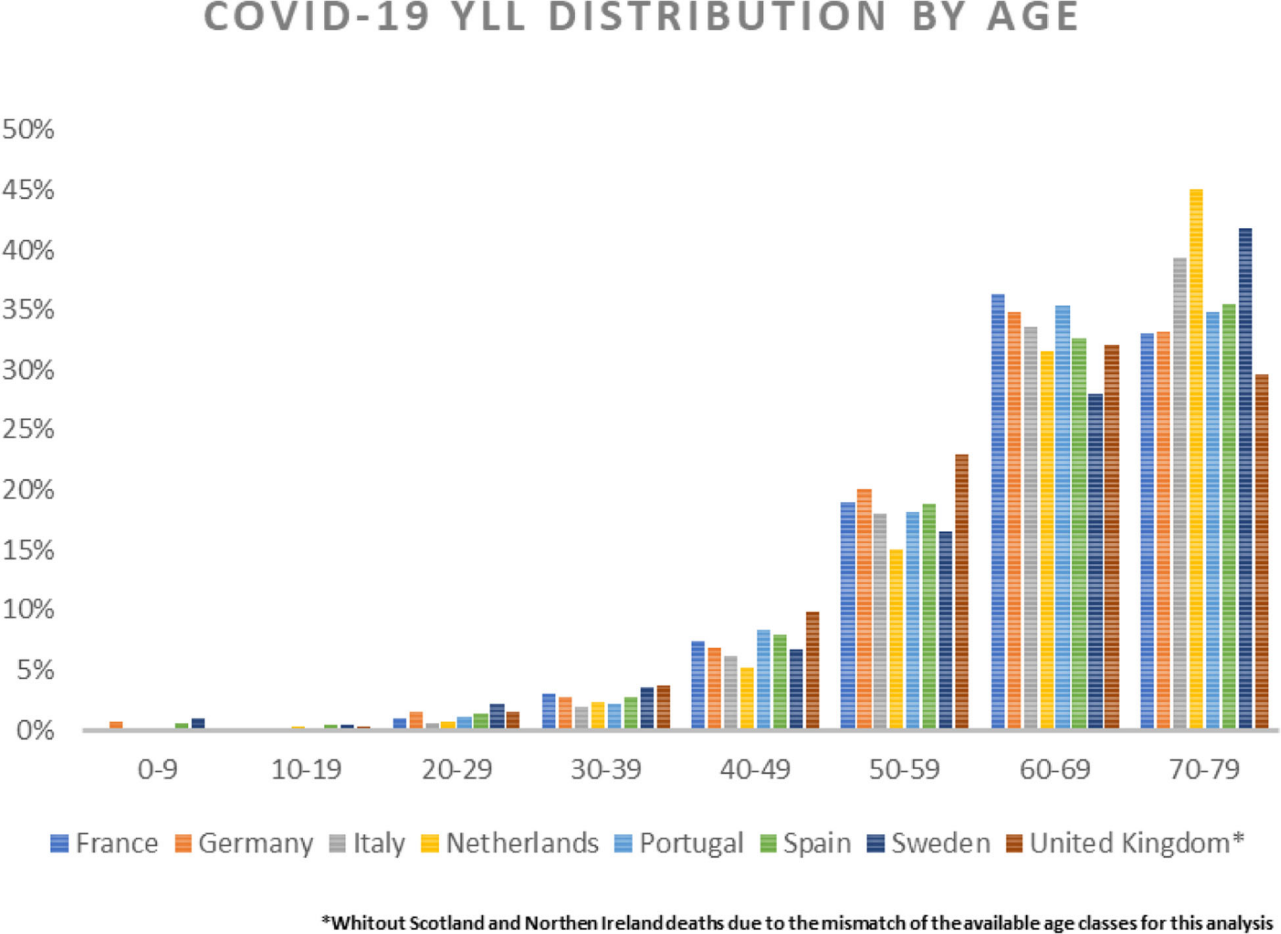
Distribution of YLL by COVID-19 by age in selected countries

COVID-19 affected older people disproportionately, both in terms of the death rates and YLL per 10,000 population. Deaths from COVID-19 among people aged 60+ accounted for 70% of all YLL to COVID-19. In France, those aged between 60 and 69 accounted for the largest share of YLL, followed by Portugal. In the Netherlands and Sweden, people aged 70+ accounted for the largest share of YLL to COVID-19 (Fig. 1).

COVID-19 affected men disproportionately, accounting for most of the YLL in every country considered in this study. Portugal was the country where there seemed to be more of a gender balance (Additional file 1).

### Excess YLL to COVID-19 and other causes during the pandemic in 2020

Between 2 March 2 and 27 December 2020, Portugal registered 38,308 deaths due to all causes among people aged less than 80. During that period, the country registered 2140 deaths due to COVID-19 aged less than 80, or 5.6% of all deaths. Based on the mortality rates between 2017 and 2019, we estimate that Portugal had an excess of 35,510 YLL (+ 6.2%), of which 72% would have been due to COVID-19 and 28% due to non-COVID-19 causes, respectively. Among the countries studied, COVID-19 had a greater impact in Portugal in relation to the number of excess YLL. All the European countries considered, except for Germany, experienced overall excess mortality during the first year of the pandemic.

**Fig. 2:**
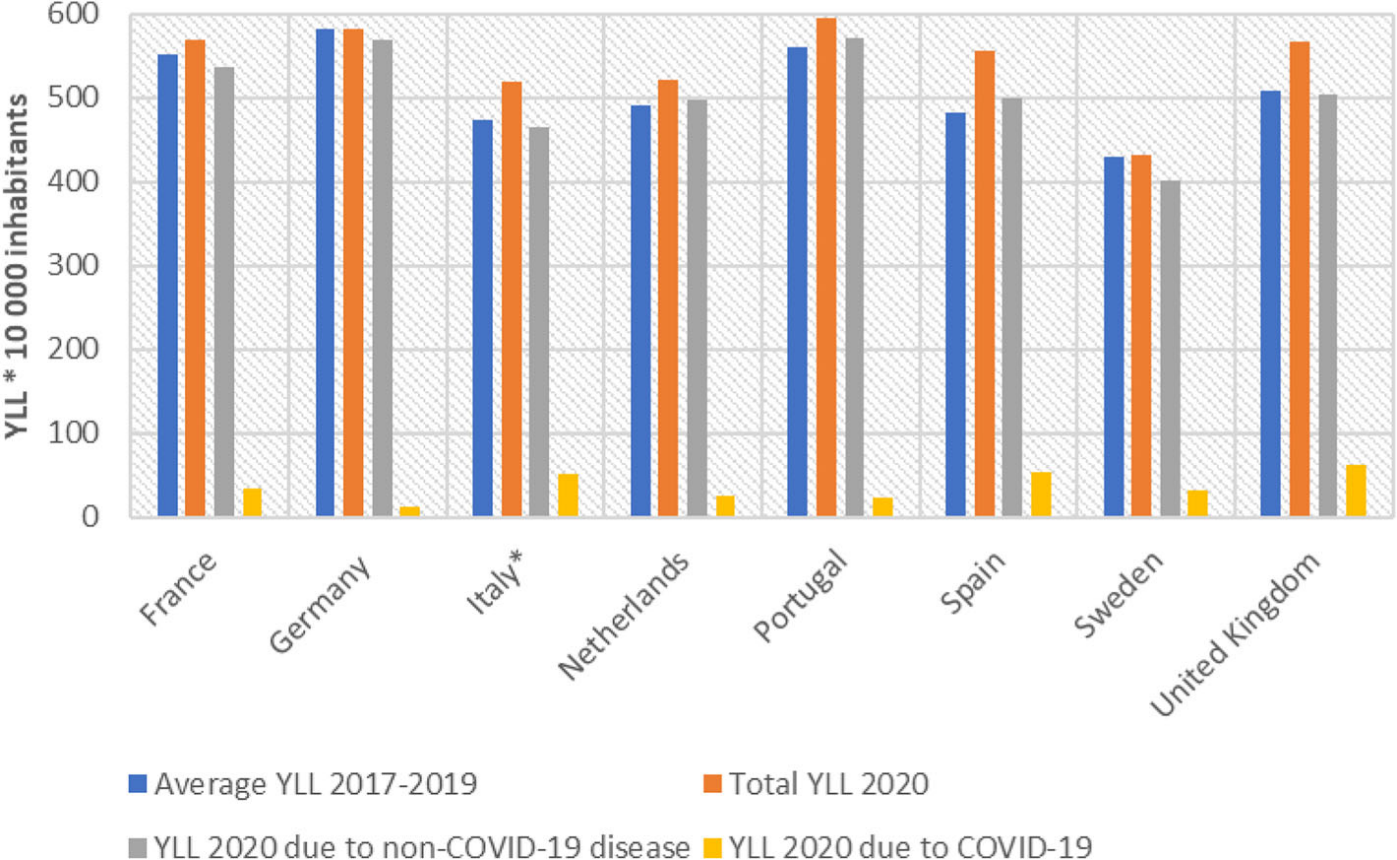
YLL by country between week 10 and week 52 of 2020 due to COVID-19 and non-COVID-19 causes

In Fig. 2, we can see that Portugal registered the highest rate of total YLL, 595 per 10,000 inhabitants in 2020, followed by Germany (582), France (569), and the United Kingdom (567). However, the United Kingdom (62), Spain (55), and Italy (53) had the highest YLL per 10,000 inhabitants directly lost by COVID-19.

The total YLL was higher in 2020 than in 2017–2019 in every country considered in this study, except for Germany, where it was nearly even (0.04%).

In addition to registering an excess YLL to COVID-19, Portugal, Spain, and the Netherlands also registered an excess YLL per 10,000 population to non-COVID-19 causes (exc-YLL-non-Cvd). In every country considered here, COVID-19 accounted for most of the excess YLL in 2020. However, Portugal reported the highest ratio (40%) between excess YLL to non-COVID-19 causes and YLL to COVID-19 (see Additional file 1 for more detail). This means that for every YLL to COVID-19, there was 0.4 YLL to non-COVID-19 causes.

No correlation was found between YLL to COVID-19 and exc-YLL-non-Cvd among countries (*r*^2^ = 0.240, *p* = 0.568).

### Estimation of excess YLL to CA, CVDs, and DRSs

Between 2012 and 2016, 235,133 people below the age of 80 died in Portugal. CA, CVDs, and DRSs contributed on average to 37.3, 22.7, and 7.3%, respectively, of all deaths from all causes in individuals below the age of 80.

For that same period, CA, CVDs, and DRSs contributed to 37.6, 17.7, and 5.2%, respectively, of all YLL among people aged less than 80 years (Additional file 1). This relative distribution was similar in all other countries in the study, with a maximum variation between countries of 9%.

CA accounts for a higher proportion of YLL in Portugal than in the average of the 28 European Union countries. Conversely, CVDs accounted for a smaller proportion of YLL in Portugal compared to that in the 28 European Union countries.

Applying the relative distribution of the YLL per cause observed in 2012–2016 to the YLL to non-COVID-19 deaths during this period of the pandemic, we estimated that the number of YLL in excess due to CA, CVDs, and DRSs from 2 March to 27 December 2020, in Portugal, would have been 3805.3, 1786.3, and 524.5, respectively (Table 2).

**Table 2:** Estimates of excess YLL to non-COVID-19 diseases

	***YLL to*** ***COVID-19***	***Excess YLL to non-COVID-19 disease***	***Excess YLL***		
			***Cancer (CA)***	***Cardiovascular diseases (CVDs)***	***Diseases of the respiratory system (DRSs)***
***France***	229,009.1	-^a^	-^a^	-^a^	-^a^
***Germany***	111,756.0	-^a^	-^a^	-^a^	-^a^
***Italy***	318,760.0	-^a^	-^a^	-^a^	-^a^
***Netherlands***	42,851.8	11,257.5	4998.9	1963.3	605.0
***Portugal***	25,394.5	10,115.0	3805.3	1786.3	524.5
***Spain***	259,059.0	85,926.47	36,608.3	15,942.6	5043.8
***Sweden***	32,356.0	-^a^	-^a^	-^a^	-^a^
***United Kingdom***	422,118.0	-^a^	-^a^	-^a^	-^a^

*YLL* years of life lost.

^a^No excess YLL to non-COVID-19 disease

## Discussion

In this paper, we estimated that Italy, the United Kingdom, and Spain had the greatest amount of YLL directly due to COVID-19 and that Spain, Portugal, and the Netherlands had excess YLL due to non-COVID-19 causes.

Few studies have tried to estimate the YLL to COVID-19 and to non-COVID-19 causes in 2020 [18, 21, 27]. This paper does not calculate the YLD and, consequently, the DALYs [12] for COVID-19 because no firm data exists (yet) for a deeper understanding of the long-term effects of thisdisease, even though some COVID-19 survivors may report histological, imagiological, and epidemiological evidence that suggests they may produce relevant burden of disease in futureyears [28–30]. The most severe forms of acute disease seem to affect mainly older people, many of whom already have significant comorbidities and disabilities, who end up dying in a few weeks. The increased disability during those weeks adds little to what is learned by calculating YLL. Thus, as the DALYs were not calculated, any value was not discounted or weighted for YLL as referenced in other methods [11].

In a recent study [18], it was found that men had about 45% more YLL to COVID-19 than women. This is close to our findings, where we state that men had around 60% of YLL to COVID-19 in the selected European countries. A higher average age-at-death for women and a higher number of affected men with COVID-19 are factors that may account for these results [18].

In the United State, it was estimated that, by early July, there were already 1.2 million YLL to COVID-19 and that by October that number already exceeded 2.5 million [31], 76.16 YLL/10000 inhabitants. This rate is much higher than that observed in this study, where the highest rate was registered in the United Kingdom (62.00 YLL/10000 inhabitants).

The assumption that the mortality by age and cause registered between 2012 and 2016 would apply to 2020 if COVID-19 had not occurred is fair. CA, CVDs, and DRSs are chronic and degenerative issues that do not change over short periods of time, except in cases of major events that drastically change the environmental, social, and economic determinants of health, as happened at the time of the breakup of the Soviet Union [32]. However, it is certainly an area to be investigated in the future.

Due to its contagiousness and seriousness, COVID-19 exerts strong pressure on the human and technical resources of the entire health system [33]. A relevant part of the pre-hospital care facilities, consultations, diagnostic facilities, general inpatient beds, and intensive care beds are occupied or reserved for patients with COVID-19 [9]. As it is impossible to increase the supply of these resources in proportion to the growth in demand, patients with other pathologies, such as CA, CVDs, DRSs, end up having their consultations, exams, hospitalizations, and scheduled surgeries postponed, sometimes sine die. It is reasonable to assume that the delay or lack of care may have had an impact on the health and survival of these patients.

The capacity of each country’s healthcare system to manage COVID-19 and non-COVID-19 patients may partly explain the difference in excess YLL to COVID-19 and other causes. Also, a lower intensity flu season in the analysed countries may be in part responsible for the lower mortality found in some nations [34]. In Portugal, as a result of the COVID-19 pandemic, there was a radical change in the availability and use of health services [9], because a significant part of the capacity of the national healthcare system was reallocated for the treatment of patients with COVID-19. In a previous report [10], we estimated an overall excess of 11,736 deaths between March and December 2020 in Portugal. This number is above the estimated excess YLL (10115) because, although they are related, they represent different outcomes. YLL only accounts for deaths below the age of 80 and increases with deaths that occur at younger ages. In this case, this is in line with the fact that most excess deaths were mainly seen at advanced ages.

Of all countries, Portugal stands out as having the highest ratio of excess YLL to non-COVID-19/COVID-19 YLL. This ratio can be a proxy for the countries capacity in coping with increased pressure on the healthcare system regarding management of non-COVID-19 disease. Some characteristics, such as the age distribution of the population, lack adherence to the policy measures during different phases of the pandemic, and lack of preparedness on behalf of healthcare systems may have contributed to these results in Portugal.

The increase of incidence of COVID-19 may end up with YLL beyond the expected values, even beyond the direct burden of this disease, which parallel to control the pandemic. Although no correlation was found in our study (probably due to organizational differences between healthcare systems between countries), countries need to be aware that exc-YLL-non-Cvd can seriously increase if there are no efforts to care for the short- and long-term management of other diseases. This is probably related to the lack of clinical follow-up, screenings, postponing surgeries, diagnostics, and healthcare-seeking hesitancy.

These results of this paper compare well with other studies, because they used the conventional methods of calculating YLL. This study focused on only eight European countries, because it was difficult to get the number of COVID-19 deaths by age and gender for many other countries. Our estimates of YLL may have some degree of error because mortality data by age and cause is available only up to 2016 in many countries and is referred to as provisory for some countries in 2020. Also, estimates of YLL are conservative, because we used 80 years of age as the upper limit for the range class [21]. These results may be even more conservative due to an expected decrease in the proportion of deaths by other non-natural causes, such as traffic accidents, increasing the proportion of deaths by other natural diseases. In fact, in a brief analysis, we found that the proportions of deaths by non-natural causes in Portugal were about 1.7, 1.6, and 1.5% in the years 2017, 2018, and 2019, respectively, and 1.3% in 2020 [35].

Results of this study must be interpreted cautiously. At first glance, it may seem obvious that countries with a higher LE are more penalized in the calculations of YLL. However, some of the characteristics that cause countries to obtain a higher average LE are also factors that contribute to better control of deaths at younger ages, such as a possible better organization and responsiveness of the National Health Systems, socioeconomic factors, and/or better health literacy levels, which can assist in the adoption of more preventive and responsible behaviours by their populations in controlling the dissemination of the virus [36, 37]. YLL is a different measure and may be very different when comparing it to overall mortality. It is dependent on the LE for each country and only for people below that value.

Other limitations are that the calculations of YLL were not adjusted by age, as some countries did not have these distributions available. As the data obtained are grouped, some analyses were not possible to be carried out, such as the calculation of confidence intervals. Also, some under-ascertainment in certification of COVID-19 deaths across Europe is not ruled out [38].

This study needs to be replicated later, as since January 2021, the number of cases and deaths of COVID-19 have greatly increased all across Europe [39].

COVID-19 has had a major impact on mortality rates in Portugal, as in other European countries, both in terms of the number of deaths and number of YLL. The relative impact of COVID-19 on the number of deaths has been greater than that on the number of YLL, because COVID-19 affects older people disproportionately. COVID-19 accounts for the overwhelming number of excess deaths and excess YLL in Portugal, more so than in other the European countries considered in this study. All the countries considered experienced overall excess YLL during the first year of the pandemic. Spain, Portugal, and the Netherlands experienced significant excess YLL to non-COVID-19 causes. COVID-19 accounted for far more YLL than the excess YLL caused by CA, CVDs, and DRSs combined.

## Conclusions

The relative impact of COVID-19 on the number of deaths has been greater than that on the number of YLL, because COVID-19 affects older people disproportionately. Estimating the number of YLL to COVID-19 is an important method to measure the burden of this disease per country. Not all countries that had more YLL to COVID-19 had a greater excess YLL to other causes. Differences between countries in the number of excess YLL to non-COVID-19 causes are related to the capacity of healthcare systems to adapt and respond to COVID-19 and non-COVID-19 diseases.

## Supplementary Information

**Additional file 1.**

## Data Availability

The datasets generated and/or analyzed during the current study are available in the datasets mentioned in the Methods section.

## Abbreviations

CA: Cancer
COVID-19: Coronavírus-19
CVDs: Cardiovascular Diseases
DALY: Disability-Adjusted Life Years
DRSs: Diseases of Respiratory System
LE: Life’s Expectancy
Exc-YLL-non-Cvd: YLL in Excess without COVID-19
YLL: Years of Life Lost
