# Supplementary material for: Years of life lost by COVID-19 in Portugal and comparison with other European countries in 2020
Source: BMC Public Health. 2021 Jun 2;21:1054. doi: 10.1186/s12889-021-11128-6 (PMC8171993; doi:10.1186/s12889-021-11128-6)
Supplement: Supplementary file 1 — Additional file 1. [file 12889_2021_11128_MOESM1_ESM.docx]

APPENDIX 1

Descriptive analysis of deaths by all-cause by age (*n*) between weeks 10 and 52 by year (Eurostat Data)

| *Year 2017* | 0-9 | 10-19 | 20-29 | 30-39 | 40-49 | 50-59 | 60-69 | 70-79 | **Total** |
| --- | --- | --- | --- | --- | --- | --- | --- | --- | --- |
| France | 3119 | 1019 | 2752 | 4824 | 12159 | 30595 | 60264 | 78990 | **193722** |
| Germany | 2646 | 981 | 2626 | 5325 | 13765 | 46834 | 88926 | 169272 | **330375** |
| Italy | 1434 | 645 | 1478 | 2657 | 8659 | 21111 | 44358 | 95811 | **176153** |
| Netherlands | 617 | 240 | 515 | 869 | 2357 | 6842 | 15197 | 27021 | **53658** |
| Portugal | 262 | 133 | 357 | 745 | 2277 | 5440 | 9554 | 17337 | **36105** |
| Spain | 1184 | 466 | 1069 | 2551 | 7598 | 19654 | 33869 | 57847 | **124238** |
| Sweden | 306 | 149 | 488 | 538 | 1089 | 2724 | 7271 | 16031 | **28596** |
| United Kingdom | 2991 | 975 | 2750 | 5662 | 12564 | 27974 | 55100 | 104606 | **212622** |

| *Year 2018* | 0-9 | 10-19 | 20-29 | 30-39 | 40-49 | 50-59 | 60-69 | 70-79 | **Total** |
| --- | --- | --- | --- | --- | --- | --- | --- | --- | --- |
| France | 3 100 | 993 | 2665 | 4954 | 11970 | 30089 | 59750 | 81967 | **192388** |
| Germany | 2 696 | 1020 | 2664 | 5329 | 13365 | 47990 | 93373 | 170364 | **334105** |
| Italy | 1 409 | 646 | 1451 | 2646 | 8211 | 21223 | 42828 | 93152 | **170157** |
| Netherlands | 630 | 210 | 554 | 891 | 2275 | 6789 | 15182 | 28072 | **54603** |
| Portugal | 317 | 138 | 319 | 671 | 2293 | 5273 | 9634 | 17842 | **36487** |
| Spain | 1 088 | 466 | 1095 | 2415 | 7468 | 19489 | 34126 | 58520 | **123579** |
| Sweden | 282 | 158 | 471 | 567 | 1042 | 2691 | 7179 | 16432 | **28822** |
| United Kingdom | 2 876 | 1070 | 3047 | 5858 | 12692 | 28498 | 54051 | 105779 | **210995** |

| *Year 2019* | 0-9 | 10-19 | 20-29 | 30-39 | 40-49 | 50-59 | 60-69 | 70-79 | **Total** |
| --- | --- | --- | --- | --- | --- | --- | --- | --- | --- |
| France | 2 990 | 1031 | 2716 | 4926 | 11452 | 29054 | 58631 | 83520 | **191330** |
| Germany | 2 588 | 945 | 2582 | 5373 | 12587 | 46215 | 92885 | 164058 | **324645** |
| Italy | 1 198 | 583 | 1435 | 2458 | 7844 | 21015 | 41728 | 91815 | **166878** |
| Netherlands | 624 | 214 | 560 | 867 | 2129 | 6671 | 14831 | 28873 | **54769** |
| Portugal | 230 | 119 | 331 | 670 | 2070 | 5174 | 9760 | 17282 | **35636** |
| Spain | 1 055 | 474 | 1031 | 2316 | 7136 | 19462 | 34158 | 59767 | **124344** |
| Sweden | 263 | 123 | 420 | 501 | 979 | 2575 | 6609 | 16269 | **27739** |
| United Kingdom | 2 778 | 939 | 3052 | 5799 | 12220 | 28391 | 53014 | 106440 | **209855** |

| *Year 2020* | 0-9 | 10-19 | 20-29 | 30-39 | 40-49 | 50-59 | 60-69 | 70-79 | **Total** |
| --- | --- | --- | --- | --- | --- | --- | --- | --- | --- |
| France | 2 660 | 1003 | 2542 | 4862 | 11677 | 30083 | 62143 | 97296 | **209606** |
| Germany | 2 553 | 859 | 2495 | 5520 | 12744 | 46597 | 96192 | 163165 | **327572** |
| Italy | 1 080 | 537 | 1244 | 2417 | 8120 | 23304 | 49142 | 111345 | **196109** |
| Netherlands | 646 | 214 | 526 | 957 | 2248 | 6820 | 15925 | 32971 | **60307** |
| Portugal | 270 | 136 | 366 | 684 | 2246 | 5524 | 10593 | 19820 | **39639** |
| Spain | 1 023 | 465 | 1064 | 2491 | 7749 | 21440 | 41158 | 76332 | **150699** |
| Sweden | 289 | 0 | 391 | 514 | 955 | 2768 | 7054 | 18448 | **30419** |
| United Kingdom | 2 519 | 873 | 2836 | 6247 | 13763 | 32987 | 61371 | 125747 | **243824** |

Descriptive analysis of deaths by COVID-19 (*n*) in 2020 (INED for all countries and by INED and NISRA for the United Kingdom (INED for Scotland, England and Wales, and NISRA for Northern Ireland)

| *Year 2020* | 0-9 | 10-19 | 20-29 | 30-39 | 40-49 | 50-59 | 60-69 | 70-79 | **Total** |
| --- | --- | --- | --- | --- | --- | --- | --- | --- | --- |
| France | 4 | 6 | 36 | 147 | 446 | 1554 | 4644 | 9592 | **16429** |
| Germany | 10 | 3 | 29 | 67 | 213 | 861 | 2436 | 6177 | **9796** |
| Italy | 7 | 6 | 31 | 128 | 517 | 2019 | 5795 | 14997 | **23500** |
| Netherlands | 0 | 2 | 5 | 21 | 60 | 239 | 800 | 2795 | **3922** |
| Portugal | 0 | 0 | 5 | 12 | 58 | 174 | 545 | 1359 | **2153** |
| Spain | 19 | 16 | 62 | 143 | 533 | 1722 | 4600 | 10943 | **18038** |
| Sweden | 4 | 2 | 12 | 24 | 58 | 193 | 515 | 1777 | **2585** |
| United Kingdom | 4 | 16 | 98 | 303 | 1031 | 3340 | 7538 | 17991 | **30321** |

APPENDIX 2

Life Expectancy between 2012 and 2016 for selected countries (PORDATA^22^).

APPENDIX 3

COVID-19 YLL Distribution by gender in different European Union countries and United Kingdom


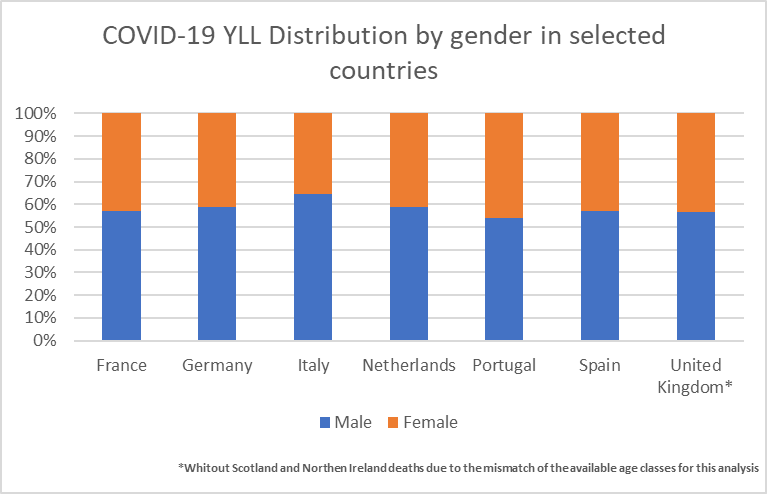


APPENDIX 4

Excess YLL in 2020 due to COVID-19 and non-COVID -19 per 10 000 inhabitants in the selected countries.


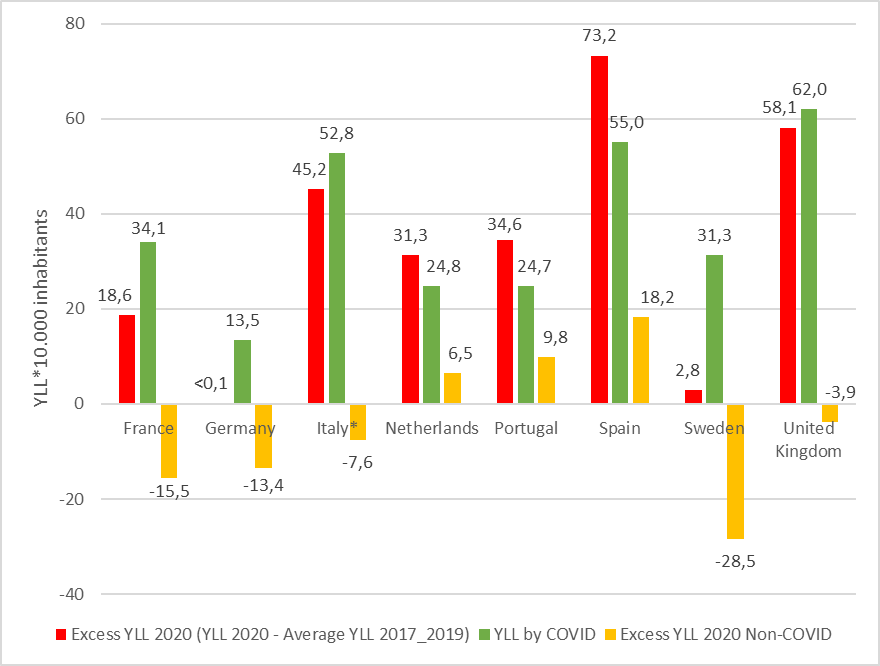


Ratio between Excess YLL due to other causes non COVID-19 and YLL directly related to COVID-19 and per country in 2020.

|  | **France** | **Germany** | **Italy** | **Netherlands** | **Portugal** | **Spain** | **Sweden** | **United Kingdom** |
| --- | --- | --- | --- | --- | --- | --- | --- | --- |
| **Ratio in 2020 (Exc YLL-Non-COVID / YLL COVID)** | **-46%** | **-100%** | **-14%** | **26%** | **40%** | **33%** | **-91%** | **-6%** |

APPENDIX 5

Distribution of average percentage of YLL by disease and Standard Deviation between 2012 and 2016 in several countries and EU28


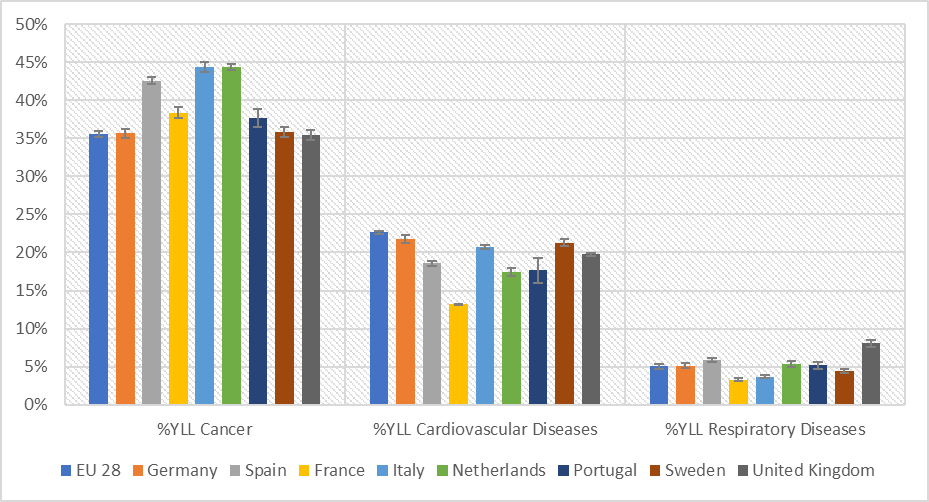


^*EU28 – The 28 Countries of European Union before 2020.^
